# Clearance of HBsAg in a patient with familial multiple myeloma after a bortezomib-based regimen combined with anti-HBV drug: A case report

**DOI:** 10.1097/MD.0000000000022642

**Published:** 2020-10-02

**Authors:** Xuzhao Zhang, Yun Liang, Xian Li, Weiqin Wang, Jiefeng Tong, Yang Xu

**Affiliations:** a Department of Hematology; b Cancer Institute (Key Laboratory of Cancer Prevention and Intervention, China National Ministry of Education), The Second Affiliated Hospital of Zhejiang University School of Medicine, Hangzhou, Zhejiang, China.

**Keywords:** bortezomib, entecavir, familial multiple myeloma, HBV clearance

## Abstract

**Rationale:**

Reactivation of hepatitis B virus (HBV) after treatment with bortezomib-based regimens in HBV-positive patients with multiple myeloma (MM) has been reported in the past few years. Nevertheless, there is evidence of inhibition of HBV replication by bortezomib in transgenic mice. However, there is still no clinical evidence that bortezomib inhibits HBV.

**Patient concerns:**

A 55-year-old MM patient with a family history of MM, who was also a chronic HBV carrier, achieved HBV clearance after treatment with a bortezomib-based regimen in combination with anti-HBV drugs.

**Diagnoses:**

The diagnosis was MM with chronic carrier of HBV.

**Interventions:**

He received bortezomib-based regimen for MM as well as entecavir as a prophylaxis to prevent HBV reactivation.

**Outcomes:**

This patient achieved HBsAg and HBV-DNA clearance after 2 months and the remission was maintained during the next 2 years. He also achieved complete remission of MM and underwent consolidation therapy with autologous hematopoietic stem cell transplantation.

**Lessons:**

This is the first case of MM with HBV clearance after receiving a bortezomib-based regimen combined with anti-HBV drug. Research on related mechanisms might provide new suggestions and hope for better management of HBV positive patients with MM and for the treatment of HBV patients.

## 1 Introduction

In East Asia, chronic hepatitis B virus (HBV) infection is often seen in patients with malignancies including multiple myeloma.[^1^,^2^,^3^] There is a risk of reactivation of HBV during the treatment of various tumors including multiple myeloma.[^4^,^5^] With the emergence of new drugs, the treatment of multiple myeloma has now entered the era of targeted therapy from the era of traditional chemotherapy. It has been reported that treatments based on new drugs including bortezomib can also lead to reactivation of HBV.[^6^,^7^,^8^,^9^,^10^] However, some studies reported that 1 mg/kg of bortezomib could decrease the copies of HBV in transgenic mice in vivo,^[11]^ although clinical evidence about the same in humans is still lacking. We report here the case of an HBV-positive patient with familial multiple myeloma, who achieved HBV clearance after treatment with a bortezomib-based regimen combined with anti-HBV drug. To our knowledge, this is the first case of HBV clearance after treatment with a bortezomib-based regimen combined with anti-HBV drug in patient with multiple myeloma.

## 2 Case report

A 55-year-old man was diagnosed with “lumbar muscle strain” at a local hospital after complains of low back pain for 3 months. However, no significant improvement was seen after symptomatic treatment (specific treatment unknown). Two months later, the patient developed back pain with bilateral pain in the rib area. On April 21, 2018, enhanced chest computed tomography (CT) showed a small amount of chronic inflammatory and fibroproliferative lesions in the lower lobe of both lungs; a small amount of effusion on both sides of the thoracic cavity, osteoporosis of the vertebrae, ribs, and sternum with multiple bone resorption and destruction, consider plasmacytoma possible. Blood biochemical tests suggest elevated globulin (88.2 g/L).

When the patient visited our hospital, there were no obvious positive findings on physical examination, except for pale lips, conjunctiva, and nail bed. Laboratory findings were as follows: white blood cell count, 3400 cells/μL; hemoglobin, 86 g/L; platelets,118,000 cells/μL; total protein, 113 g/L; albumin, 28 g/L; creatinine, 58 μmol/L; calcium, 2.24 mmol/L; β2-microglobin, 2.84 mg/L. Immunoglobulin G (IgG) was 66.79 g/L, but IgA and IgM were below normal levels, blood lambda light chain was 19.8 g/L, and the ratio of kappa/lambda was 0.01. Immunofixation electrophoresis showed an increase in monoclonal IgG and lambda in the blood. Bone marrow smear suggested elevated plasma cells (plasma cells 23%). Fluorescence in situ hybridization (FISH) results of CD138 monoclonal antibody sorting cells showed 8% positive for immunoglobulin heavy chain (IGH) rearrangement and 10% positive for 13q deletion. The diagnosis was multiple myeloma [subtype IgG/lambda Durie-Salmon stage IIIA, International Staging System (ISS) stage II]. This patient was a known carrier of HBV for more than 20 years. His elder brother had also been diagnosed with multiple myeloma (subtype IgG/kappa) in 2008 at the age of 56 years. Before initiation of treatment for multiple myeloma, the patient was seropositive for HBsAg (39 IU/mL), anti-HBe, and anti-HBc. Alanine aminotransferase (ALT) was 64 U/L (normal range <45 U/L) and aspartate aminotransferase (AST) was 69 U/L (normal range <50 U/L). Serum HBV-DNA was 161 IU/mL.

The patient received a 3-week cycle of PCD (Bortezomib 2.3 mg and Cyclophosphamide 0.4 g on day 1,4,8,11+ Dexamethasone 20 mg on day 1–2, 4–5, 8–9, 11–12). Entecavir was administered as a prophylaxis against HBV reactivation, and HBV-DNA was monitored since the start of PCD therapy. The patient tolerated the chemotherapy regimen well, and there were no obvious adverse effects. After 1 cycle of therapy, the serum HBV-DNA was undetectable. HBsAg turned negative after 2 months and was also negative during the 2 years of follow-up. However, anti-HBe and anti-HBc were still positive. After 4 courses of PCD therapy, the patient's immunofixation electrophoresis turned negative, and the free light chain returned to normal, indicating a stringent complete response (sCR). Subsequently, his peripheral blood hematopoietic stem cells (HSCs) were collected and he was consistently treated with high-dose chemotherapy followed by autologous HSC transplantation as consolidation treatment, 10 months after the initial diagnosis of multiple myeloma. The patient continues to remain in sCR at 26 months of follow-up. During subsequent treatment and follow-up, HBsAg continued to be negative and HBV-DNA continued to fall below the detection limit.

## 3 Discussion

Bortezomib is a proteasome inhibitor that is currently used in the treatment of multiple myeloma, mantle cell lymphoma, Waldenström macroglobulinemia/lymphoplasmacytic lymphoma, and other plasma cell-related diseases. The rate of reactivation of the herpes zoster virus was significantly higher in patients receiving bortezomib,^[12]^ suggesting that bortezomib might cause reactivation of the virus. In East Asian countries including China, the proportion of multiple myeloma patients who are positive for HBV infection ranges from 3.4% to 19.4%.[^1^,^2^,^13^] In recent years, HBV reactivation has been reported in HBV-positive patients of multiple myeloma, after receiving bortezomib-based regimens.[^6^,^7^,^8^,^9^,^10^] However, bortezomib-based regimens usually contain glucocorticoid, which has a negative effect on HBV infection,^[14]^ and maintenance therapy with prednisolone can also result in HBV reactivation in patients of multiple myeloma.^[15]^ Moreover, studies have shown that autologous HSc transplantation is one of the important risk factors for HBV reactivation.[^16^,^17^] These factors also influence the assessment of the role of bortezomib in HBV reactivation. More recently, a study by Hou et al^[18]^ revealed that bortezomib might promote HBV activation by inhibiting ubiquitination degradation of HBV polymerase. However, proteasome inhibitor was reported to inhibit HBV production in vitro,^[19]^ and in vivo. Studies conducted on transgenic mouse model have found that bortezomib can inhibit HBV replication.^[11]^ However, there is still no clinical evidence to prove that bortezomib can inhibit HBV.

This case is unique in 2 ways: first, the patient and his elder brother were diagnosed with multiple myeloma at a similar age; second, this patient achieved HBsAg clearance after receiving bortezomib-based regimen combined with anti-HBV drug. To our knowledge, this is the first case report about HBsAg clearance following bortezomib-based regimen combined with entecavir as antiviral therapy.

Clearance of HBsAg is one of the important signs of HBV clearance. Possible causes of achieving HBsAg negativity include elimination of HBV and mutation of HBsAg antigen gene, due to which HBsAg is not expressed.^[20]^ In this case, at the same time that the patient's HBsAg turned negative, the HBV-DNA was also undetectable, which ruled out the possibility of HBsAg gene mutation leading to HBsAg clearance. Although the probability is low, spontaneous clearance of HBsAg still occurs in some HBsAg-positive patients.[^21^,^22^] However, this patient did not achieve spontaneous clearance of HBsAg in the past 20 years. Therefore, it is likely that this patient achieved HBsAg clearance by combined treatment with bortezomib and entecavir, rather than spontaneous clearance. However, some studies have shown that about 20% of the patients develop HBV reactivation 2 years after initiation of bortezomib treatment,^[23]^ even in patients with resolved HBV infection.^[1]^ Therefore, it remains to be seen whether HBV clearance will be maintained in this patient in the future.

HBsAg clearance is related to many factors, including HBV subtypes, treatment strategies, and certain genetic polymorphisms in patients.[^24^,^25^,^26^,^27^,^28^] HBsAg level is one of the important predictors of HBV clearance after antiviral treatment^[29]^; therefore, low serum HBsAg levels in this patient might be a precursor for HBV clearance. Zhang et al^[30]^ showed that bortezomib did not affect the replication of the wild-type HBV virus, while increasing the replication of X-negative HBV virus in vitro. This suggests that alterations in the HBV genome might also influence the effects of bortezomib on HBV. Patients of multiple myeloma patients are usually immunocompromised, which can also lead to reactivation of HBV.^[31]^ The multiple myeloma in our patient was quickly controlled after initiating the PCD regimen and the improved immune status might also be one of the factors aiding the clearance of HBV. The effect of bortezomib on HBV is influenced by many factors; however, the detailed mechanism is still not understood.

Multiple myeloma is thought to have an inherited genetic component that might have existed about a century ago. Eight families with 2 or more first-degree relatives with multiple myeloma have been reported in 1974.^[32]^ Several systematic epidemiological family studies confirmed that first-degree relatives of patients with multiple myeloma have about 2 to 4 times higher risk of developing multiple myeloma.[^33^,^34^] Recently, several Genome-Wide Association Studies (GWAS) studies have identified some DNA sequence variants at an independent locus that is associated with the risk of multiple myeloma.[^35^,^36^,^37^] This patient and his elder brother were diagnosed with multiple myeloma at a similar age. Unfortunately, his brother died of multiple myeloma 3 years ago, and the exact genetic risk factors could not be determined. Our patient had a hereditary risk of multiple myeloma, and it is unclear whether some unknown genetic change could affect the outcome of his anti-HBV treatment. Whether there is a correlation between the genetic risk factors for multiple myeloma and the outcome of anti-HBV treatment needs further research.

Antiviral prophylaxis for HBV reactivation is recommended during the treatment of HBV patients with hematological malignancies, including multiple myeloma. Lu et al^[2]^ showed that bortezomib combined with antiviral prophylaxis did not lead to HBV reactivation or abnormal liver functions. Therefore, it is safe to use prophylactic antiviral therapy in HBV positive patients with multiple myeloma who receive bortezomib-based therapy. Preventive antiviral treatment is feasible, both from the perspective of preventing HBV reactivation and in aiding clearance of the HBV infection. Clinical trials should be conducted in the future to verify whether bortezomib combined with anti-HBV drugs can clear HBV infection.

## 4 Conclusion

This case suggests that anti-HBV virus treatment while receiving bortezomib therapy might achieve clearance of HBV infection in some HBV-positive patients with multiple myeloma. However, this hypothesis needs further validation. Further research is necessary to understand the detailed mechanism. These mechanisms can bring a new hope for the treatment of HBV infection.

## Acknowledgments

We thank Dr. Jinfan Li, from the Department of Pathology the Second Affiliated Hospital of Zhejiang University School of Medicine, for the kind help in pathology diagnosis and drafting the manuscript.

## Author contributions

ZX and LY designed the study, ZX, LX, WW, TJ, and XY collected the data, ZX and XY wrote the manuscript. All authors have read and approved the final manuscript.
